# d-Mannose Treatment neither Affects Uropathogenic *Escherichia coli* Properties nor Induces Stable FimH Modifications

**DOI:** 10.3390/molecules25020316

**Published:** 2020-01-13

**Authors:** Daniela Scribano, Meysam Sarshar, Carla Prezioso, Marco Lucarelli, Antonio Angeloni, Carlo Zagaglia, Anna Teresa Palamara, Cecilia Ambrosi

**Affiliations:** 1 Department of Public Health and Infectious Diseases, Sapienza University of Rome, 00185 Rome, Italy; daniela.scribano@uniroma1.it (D.S.); carla.prezioso@uniroma1.it (C.P.); carlo.zagaglia@uniroma1.it (C.Z.); 2 Dani Di Giò Foundation-Onlus, 00193 Rome, Italy; 3 Department of Public Health and Infectious Diseases, Sapienza University of Rome, Laboratory Affiliated to Institute Pasteur Italia-Cenci Bolognetti Foundation, 00185 Rome, Italy; meysam.sarshar@uniroma1.it (M.S.); annateresa.palamara@uniroma1.it (A.T.P.); 4 Microbiology Research Center (MRC), Pasteur Institute of Iran, Tehran 1316943551, Iran; 5 Department of Experimental Medicine, Sapienza University of Rome, 00185 Rome, Italy; marco.lucarelli@uniroma1.it (M.L.); antonio.angeloni@uniroma1.it (A.A.); 6 Pasteur Institute Cenci Bolognetti Foundation, 00161 Rome, Italy; 7 IRCCS San Raffaele Pisana, Department of Human Sciences and Promotion of the Quality of Life, San Raffaele Roma Open University, 00166 Rome, Italy

**Keywords:** uropathogenic *E. coli*, CFT073, d-mannose, FimH, urinary tract infections (UTIs), bacterial sugar metabolism

## Abstract

Urinary tract infections (UTIs) are mainly caused by uropathogenic *Escherichia coli* (UPEC). Acute and recurrent UTIs are commonly treated with antibiotics, the efficacy of which is limited by the emergence of antibiotic resistant strains. The natural sugar d-mannose is considered as an alternative to antibiotics due to its ability to mask the bacterial adhesin FimH, thereby preventing its binding to urothelial cells. Despite its extensive use, the possibility that d-mannose exerts “antibiotic-like” activity by altering bacterial growth and metabolism or selecting FimH variants has not been investigated yet. To this aim, main bacterial features of the prototype UPEC strain CFT073 treated with d-mannose were analyzed by standard microbiological methods. FimH functionality was analyzed by yeast agglutination and human bladder cell adhesion assays. Our results indicate that high d-mannose concentrations have no effect on bacterial growth and do not interfere with the activity of different antibiotics. d-mannose ranked as the least preferred carbon source to support bacterial metabolism and growth, in comparison with d-glucose, d-fructose, and l-arabinose. Since small glucose amounts are physiologically detectable in urine, we can conclude that the presence of d-mannose is irrelevant for bacterial metabolism. Moreover, d-mannose removal after long-term exposure did not alter FimH’s capacity to bind to mannosylated proteins. Overall, our data indicate that d-mannose is a good alternative in the prevention and treatment of UPEC-related UTIs.

## 1. Introduction

Urinary tract infections (UTIs) are among the most common infectious diseases, affecting more than 150 million people worldwide each year [1,2]. Clinically, UTIs are distinguished into cystitis or lower UTI, affecting the bladder, and pyelonephritis or upper UTI, involving the kidneys [3,4,5]. Cystitis affects mainly women due to anatomic differences of their urogenital system; it has been estimated that more than 50% of women experience at least one UTI in their lifetimes [6,7,8]. Infecting pathogens might cause asymptomatic or symptomatic infections, characterized by irritative and painful symptoms [9]. In the majority of cases, uropathogenic *Escherichia coli* (UPEC) might originate from the intestinal microbiota through the bacterial migration to the perianal region and, therefore, to the urinary tract [10]. Among the wide range of bacterial pathogens associated to UTIs, UPEC are predominant in both symptomatic and/or asymptomatic infections, accounting for up to 75% to 95% of reported UTIs [4,10,11]. While UPEC possess a plethora of well-studied virulence factors, the ability to adhere to host epithelial cells is crucial for the establishment and progression of the infection [1,4,10,11]. This ability relies mostly on type 1 fimbriae which are characterized by the presence of an adhesin, FimH, located at the tip of these fimbriae [12]. FimH binds mainly to terminal epitopes of high mannosylated glycans conjugated to uroplakin 1a (UP1a), a receptor specifically expressed on the surface of urothelial cells, known as the catch–bond binding mechanism [5,8,13,14,15]. Therefore, being abundantly distributed on the majority of UPEC strains, type 1 fimbriae are the main appendages responsible for the bacterial adhesion to bladder epithelial cells [16,17].

Diagnosis of UTIs is based on clinical signs as well as urine tests to identify the infecting microorganism(s) and its antimicrobial susceptibility profile [9,10]. However, despite appropriate and often successful antibiotic treatments, around 20−40% of women experience at least one recurrence within six months of their initial diagnosis [2,18]. Recurrence is characterized by relapse in clinical symptoms and could be associated to persistence of bacteria that cause the primary infection or re-infection. Recurrent UTIs (rUTIs) have a profound impact on quality of life and cause a significant economic burden [1,4,10,18]. It has been recently demonstrated that UPEC isolates from elderly patients with rUTI were cell-wall deficient (l-form) bacteria. This feature makes bacteria resistant to cell wall-targeting antibiotics, likely contributing to the recurrence of the infection [19]. Moreover, the increase in antibiotic resistance found in clinical UPEC isolates has made UTI management progressively costlier and more challenging [5,10,18]. On this basis, several promising efforts have been made to study novel strategies aimed at specifically counteracting bacterial virulence factors without affecting bacterial lifestyle, metabolism, or multiplication. These kinds of approaches might be able to prevent bacterial pathogenic effects without inducing the selection of resistant strains, an unsought consequence of antibiotic treatments. For this reason, molecules able to interfere with bacterial virulence mechanisms have been proposed to combat UPEC infections [20]. Accordingly, FimH antagonists, such as d-mannose and its derivatives, have emerged as anti-virulence therapeutic strategies for the treatment of UTIs [21,22,23,24,25,26,27]. d-mannose, a C-2 epimer of d-glucose, as well as d-mannose-analogs, prevent FimH-mediated bacterial adhesion through a competitive inhibition mechanism [18,21,22,24,28]. This mechanism is based on the structural similarity between d-mannose and urothelial mannosylated receptors exposed by the epithelium of the urinary tract. When it is administered in sufficient amounts, d-mannose is rapidly absorbed and then excreted by the urinary tract where it saturates bacterial FimH, thereby preventing its binding to urothelia [29,30]. Thus, the d-mannose–UPEC interaction facilitates the clearance of bacteria that are dragged by the flow of urine [28]. The first evidence of d-mannose effectiveness in preventing UTIs was demonstrated in animals in 1979 but, thereafter, many others have been collected in humans [31,32,33,34,35]. While validated in only three clinical trials studies, d-mannose, administered at high dosage, revealed its efficacy both in reducing the symptoms and the recurrence rate of UTIs [31,34,36]. Despite these promising results, the possibility that d-mannose exerts only anti-adhesive activity on FimH without altering bacterial growth and/or metabolism not have been addressed yet. Therefore, our study aimed to investigate whether d-mannose might affect bacterial shape, viability, motility, or metabolic activity of the prototype UPEC strain CFT073. We also evaluated whether d-mannose removal after a prolonged exposure might induce mutations on FimH critical residues, thereby stably modifying FimH’s ability to bind to human mannosylated proteins.

## 2. Results and Discussion

### 2.1. d-mannose Does Not Affect Bacterial Phenotype or Sensitivity to Antimicrobials

**Figure 1:**
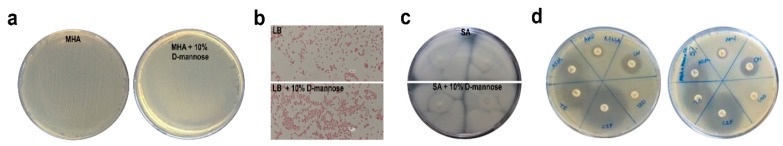
Impact of d-mannose on bacterial properties and antimicrobial activity. (**a**) Strain CFT073 was seeded on Mueller Hinton agar (MHA) plates supplemented with and without 10% d-mannose. (**b**,**c**) Otherwise, strain CFT073 grown overnight in Luria Bertani (LB) broth with and without 10% d-mannose was Gram stained or spotted onto soft agar (SA) plates, respectively. (**d**) The antibiotic susceptibility of strain CFT073 was assessed in the presence or absence of 5% d-mannose by the Kirby–Bauer disk diffusion susceptibility test on MHA plates; CN: Gentamicin; AMP: Ampicillin; AZM: Azithromycin; LZD: Linezolid; TE: Tetracycline; CIP: Ciprofloxacin. Representative images of two independent experiments.

To evaluate whether d-mannose might affect bacterial growth, strain CFT073 was streaked on Mueller Hinton agar (MHA) plates supplemented with diverse sugars at different concentrations (d-mannose, d-glucose, d-fructose, and l-arabinose). No differences in bacterial growth were observed for d-mannose concentrations up to 10% (Figure 1a; data not shown). Above this concentration, all the tested sugars reduced or inhibited bacterial growth due to the osmotic pressure. Considering that the range of d-mannose dosage for UTI prevention used in clinical trials was between 2 and 3 g per day and that the normal urine volume is 800 to 2000 mL/day, it is reasonable to speculate that d-mannose concentration ranges between 0.10 and 0.25% per day in urine, 40–100-fold less than the concentration strain CFT073 can tolerate. Moreover, no differences in bacterial morphology or motility were detected in d-mannose incubated bacteria in comparison to untreated bacterial cells (Figure 1b,c). A slight degree of d-mannose-mediated bacterial aggregation was visible, as previously reported for *E. coli* cells treated with d-mannose functionalized substrates [37,38,39].

Since sugars can influence the activity of conventional antibiotics [40,41], a Kirby–Bauer susceptibility test using strain CFT073 was performed. No difference in the size of halos was observed in MHA plates with or without d-mannose (Figure 1d), thereby ruling out any d-mannose influence on the antibacterial activity of several classes of antibiotics.

### 2.2. In the Hierarchy of Sugar Used by the UPEC Strain CFT073, d-mannose Is Ranked Lowest

**Figure 2:**
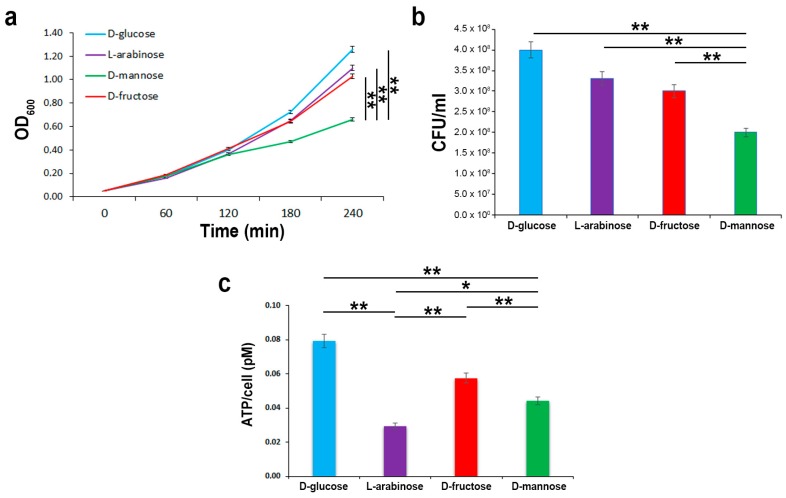

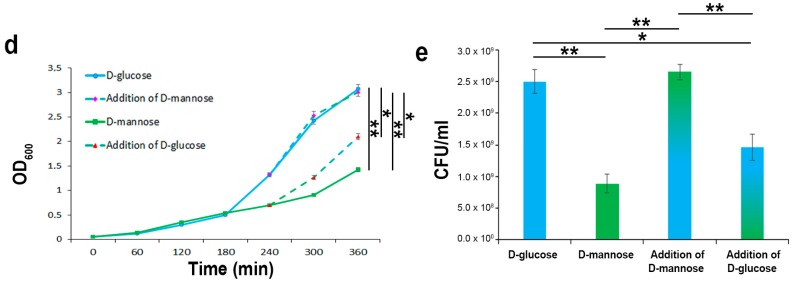
*E. coli* growth kinetics and ATP yields using different carbon sources. Strain CFT073 was grown on an M9 medium supplemented with the indicated sugars at a final concentration of 0.5% for 4 h. Growth curves were followed by OD_600_ measurements (**a**), while CFU/mL counting (**b**) and ATP intracellular content were assessed at endpoint (**c**). Strain CFT073 was grown on M9 medium supplemented with 0.5% d-glucose or 0.5% d-mannose. After 4 h, 0.5% of d-mannose or 0.5% d-glucose were added to the d-glucose or d-mannose-supplemented bacterial cultures, respectively, and the growth rates were recorded for a further 2 h by OD_600_ measurements (**d**) and CFU/mL counting at endpoint (**e**). Data represent the mean of three independent experiments. Asterisks indicate *p* values evaluated by one-way ANOVA; ** *p* ≤ 0.01, * *p* ≤ 0.05.

To evaluate the extent of d-mannose utilization in supporting bacterial growth in comparison to other sugars, strain CFT073 was inoculated in minimal M9 medium supplemented with d-mannose, d-glucose, d-fructose, or l-arabinose at a final concentration of 0.5%. The bacterial growth was monitored by optical density (OD_600_) over the course of 4 h and measured by CFU/mL at endpoint. Bacterial growth curves showed that all sugars, when administered alone, were able to sustain the growth of strain CFT073. The highest *E. coli* growth rates were recorded using the M9 medium supplemented with d-glucose, followed by both l-arabinose and d-fructose (Figure 2a,b). The lowest growth rates were obtained when d-mannose was the only carbon source in the growth medium (Figure 2a,b). In addition, the net ATP production from the metabolism of d-glucose, d-mannose, d-fructose, and l-arabinose was assessed. As expected, d-glucose provided the maximal ATP yield compared with the other tested sugars (Figure 2c). d-fructose and d-mannose were shown to be the next in terms of energy storage, whereas l-arabinose was the least (Figure 2c). It is known that d-fructose (at concentrations above 2 mM) and d-mannose are translocated across the inner membrane via the membrane-associated uptake system for mannose, encoded by the *manXYZ* operon [42]. Therefore, the lower amount of ATP formation could be due to a lesser intake of these sugars or to the energy cost for their uptake. Moreover, the low ATP yield recorded in strain CFT073 grown in an l-arabinose supplemented medium could be explained by ATP consumption. *E. coli* possesses low and high affinity l-arabinose transport systems, encoded by the *araE* and *araFGH* gene products, respectively; in addition, four genes, *araABCD*, encode for proteins required for the conversion of l-arabinose to d-xylulose-5-phosphate, which enters the pentose phosphate route [43]. Since protein synthesis is the most energy-consuming process in the cell, with more than 50% of the ATP consumption for biosynthetic purposes [44], the metabolic burden of l-arabinose could account for the lowest ATP formation. To evaluate whether *E. coli* starts to utilize d-mannose when d-glucose is in low concentrations, but not totally depleted, strain CFT073 was cultivated in M9 medium supplemented with d-glucose for 4 h before adding d-mannose to the growth medium (Figure 2d,e). Addition of d-mannose to cells growing exponentially on d-glucose as carbon source resulted neither in a temporary growth arrest nor in a statistically significant difference in the growth curves or CFU/mL with respect to bacteria grown only in d-glucose (Figure 2d,e). Vice versa, when strain CFT073 was grown in M9 medium supplemented with d-mannose and d-glucose was added after 4 h, a significant switch to this latter preferential sugar was detectable in the growth curve (Figure 2d,e). These results are in line with the knowledge that d-glucose is the preferred carbon source for *E. coli* as well as for many bacteria [45]. Therefore, even in d-glucose shortage and d-mannose abundance, *E. coli* cells still grew on their preferred sugar until it ran out, before switching to another available carbon source. The net *E. coli* preference for d-glucose is sustained by the d-glucose regulatory mechanisms that significantly inhibit the uptake of other sugars, a well-documented phenomenon referred to as carbon catabolic repression (CCR) [45]. Taken together, our data indicate that, in d-glucose deficiency, a second hierarchy in bacterial growth rates was shown encompassing d-fructose/l-arabinose followed by d-mannose. The usage of d-fructose over d-mannose to sustain bacterial multiplication could be explained in terms of energy production. Instead, l-arabinose utilization over d-mannose was quite unexpected, since l-arabinose enters the pentose phosphate pathway, which is generally believed to generate primarily pentoses and NADPH rather than ATP [46].

### 2.3. In the Presence of d-glucose, Only Basal Expression of the ManX Permease Occurs

**Figure 3:**
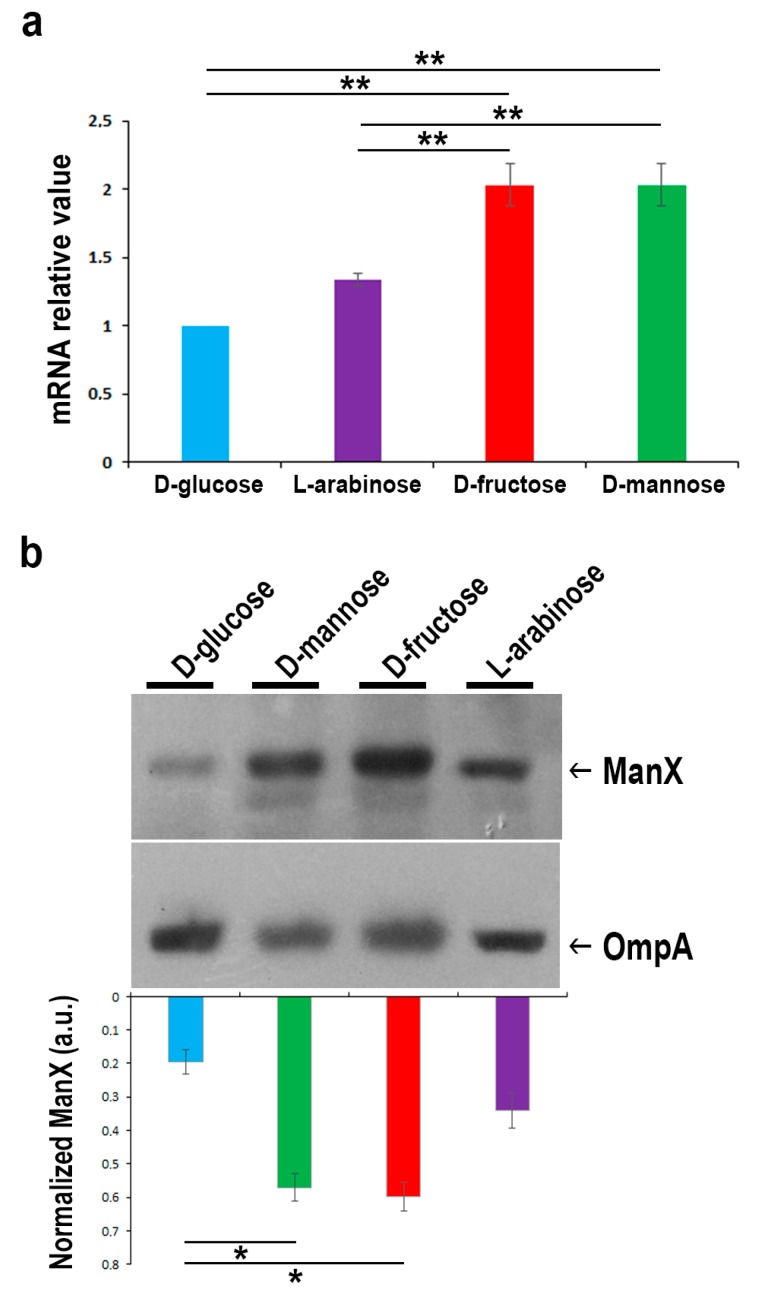
Comparison of *manX* gene and protein expression levels in strain CFT073 grown on M9 medium supplemented with 0.5% of the indicated sugars. (**a**) Real time-PCR analysis of *manX* mRNA. Total bacterial RNA was isolated from strain CFT073 grown to the mid-exponential phase, as described in the “Materials and Methods” section. The 16S rRNA gene (*rrsG*) was used as the normalizer. Data shown are fold increase (± SD) in *manX* mRNA relative to the d-glucose sample, considered 1. (**b**) Western blot analysis of ManX. Total proteins from bacteria under the same experimental conditions were separated by SDS/PAGE, transferred onto nitrocellulose, and probed with monoclonal anti-ManX antibody. The OmpA protein was used as the normalizer. Bars represent the amount of ManX calculated by densitometric analyses and expressed as arbitrary units (a.u.). Data represent the mean of three independent experiments. Asterisks indicate *p* values evaluated by one-way ANOVA; ** *p* ≤ 0.01, * *p* ≤ 0.05.

To evaluate the expression of the membrane-associated uptake system for mannose, the expression levels of the ManX permease were measured by semi-quantitative real time-PCR (RT-PCR) and Western blot in strain CFT073 grown in M9 medium supplemented with d-glucose, d-fructose, d-mannose, or l-arabinose (Figure 3). Data of the semi-quantitative analysis are reported as fold change relative to the expression of *manX* in bacteria grown in d-glucose. The expression of *manX* was twofold up-regulated in strain CFT073 cultivated in the presence of d-mannose and d-fructose, whereas no significant *manX* up-regulation was observed for those grown with l-arabinose as the unique carbon source (Figure 3a). The expression of the ManX protein was also evaluated under the same experimental conditions. Protein loading was normalized using an antibody raised against the *E. coli* outer membrane protein A (OmpA) (Figure 3b). Results confirmed that ManX expression was induced only in the presence of both d-mannose and d-fructose in the growth media since they share the same membrane transport components [47]. Conversely, only basal-level of ManX expression was detected when d-glucose was available. This result is in line with the knowledge that glucose has a plethora of constitutive uptake pathways. Indeed, glucose uptake occurs through several specific and alternate transport systems located in the cytoplasmic membrane, such as the phosphotransferase systems (PTS) for glucose, mannose, fructose, sucrose, the proton symport GalP, as well as the binding protein dependent transport (Mgl) system for galactose [48,49]. Overall, these results highlight that d-glucose is ranked at the top in a hierarchy of carbon source preference by *E. coli*, allowing for faster growth rates, whereas d-mannose is ranked lowest. We then wondered whether glucose concentration in urine from healthy individuals could be enough to prevent d-mannose utilization, which can be as high as 1–2.5 g/L in a clinical regimen of 2–3 g per day [31,34,36]. However, only a few data in the literature reported that the average amount of glucose in urine is about 0.1–120 mg/L in healthy people [50,51]. For this purpose, fifty-four urine samples from healthy subjects were tested for glucose amounts by enzyme-linked immunosorbent assay (ELISA) and showed an average concentration of 7 mg/L. Therefore, the conclusion may be drawn that *E. coli* during infection has enough availability of glucose in the bladder to sustain its metabolism and, thus, the high administered dosage of d-mannose for prevention and treatment of UTIs is irrelevant for bacterial metabolism and growth.

### 2.4. Prolonged d-mannose Treatment Does Not Induce Modifications on FimH Adhesive Properties

**Figure 4:**
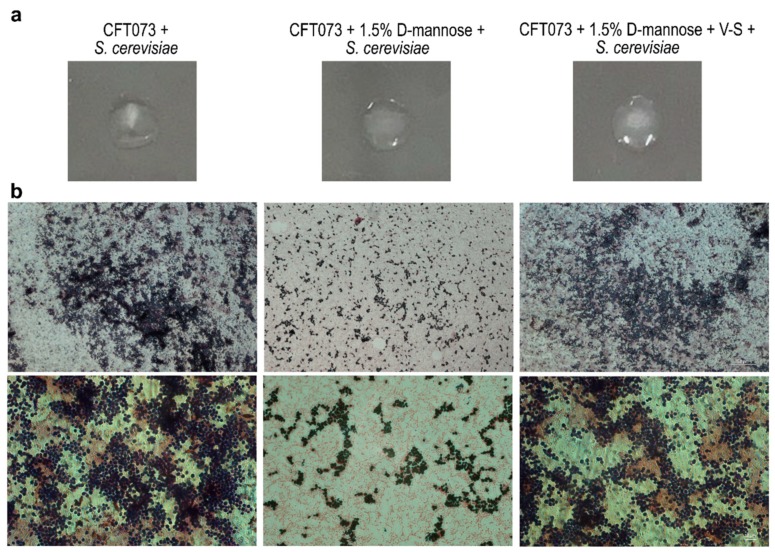
Representative images of yeast agglutination activity of *E. coli* after removal of d-mannose from FimH. Strain CFT073 grown in the presence of d-mannose was vortexed and sonicated to break FimH–d-mannose bonds (referred to as V-S bacteria). Thus, *E. coli* suspensions of untreated, d-mannose-treated, and d-mannose-treated V-S bacteria were tested for yeast cell agglutination on microscope slides. (**a**) Macroscopic images of yeast agglutination assay. (**b**) Microscopic images of Gram-stained yeast agglutination assay recorded with the 10× (upper panels) and 40× (lower panels) objectives using a Leica DM5000B microscope and processed using the Leica Application Suite 2.7.0.R1 software (Leica). Scale bars: 100 and 10 μm.

The FimH protein is composed of the mannose-binding domain (1–156 aa) and the fimbria-incorporating pilin domain (160–273 aa), which are connected by a 3-aa interdomain linker peptide chain [52]. Crystallographic studies on the FimH mannose-binding domain revealed that a pocket formed by three loops contains key amino acids that, together with two tyrosine residues (Tyr48 and Tyr137), are involved in d-mannose-binding via hydrogen bonds and van der Waals interactions [17]. Mutations in these amino acids can severely affect mannose binding [53,54,55]. To set up a method to reverse FimH–d-mannose bonds, strain CFT073 was incubated for 48 h in static conditions to induce maximal pilus formation in the presence of 1.5% d-mannose [56,57]; thereafter, one aliquot was vortexed and sonicated (referred to as V-S bacteria). We reasoned that the application of the swirling motion (vortex) and sonication could generate mechanical forces that lead to the breakage of the hydrogen and van der Waals bonds between d-mannose and FimH. Bacterial viability and FimH adhesion ability were assessed by CFU/mL counting and yeast agglutination assay, respectively [56,57]. Vitality assessment showed no reduction in the number of V-S bacteria compared to the untreated control. For the yeast agglutination assay, bacterial suspensions of untreated, d-mannose-treated, and d-mannose-treated V-S bacteria were individually incubated with *Saccharomyces cerevisiae* cells for 1 h, before being spotted on glass slides and streaked for Gram staining (Figure 4). As expected, untreated strain CFT073 agglutinated the majority of yeast cells while d-mannose-treated bacteria did this to a lesser extent, shown macroscopically and microscopically as agglutinated and dispersed yeast cells, respectively (Figure 4). Interestingly, d-mannose V-S treated bacteria agglutinated *S. cerevisiae* cells comparably to untreated bacteria, indicating that the mechanical treatment was effective in d-mannose removal, not affecting FimH interactions with yeast mannosylated proteins (Figure 4).

**Figure 5:**
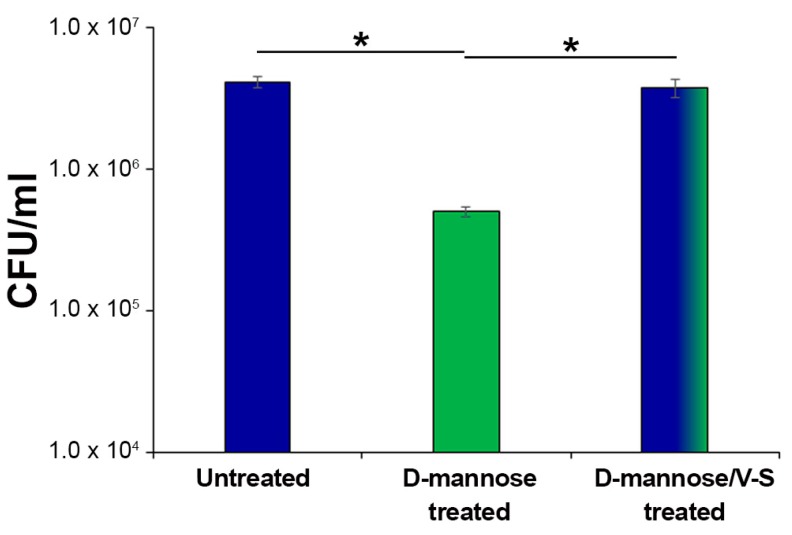
Bacterial adherence to bladder epithelial HTB-9 cells. Strain CFT073 was sub-cultured extensively with 1.5% d-mannose; one aliquot was vortexed and sonicated to break FimH–d-mannose bonds (referred to as V-S bacteria). Thus, *E. coli* suspensions of untreated, d-mannose-treated, and d-mannose-treated V-S were used to infect HTB-9 cell monolayers, at a multiplicity of infection (MOI) of 10. The total number of adherent bacteria was determined after 2 h of incubation with HTB-9 cell monolayers and expressed as CFU/mL. Data represent the mean of three independent experiments performed in triplicate. Asterisks indicate *p* values evaluated by one-way ANOVA; * *p* ≤ 0.05.

The FimH adhesin is widespread among *E. coli* and found in several natural sequence variants, characterized by different affinities for mannose residues [58]. Several point mutations were shown to favorably or detrimentally affect the affinity and specificity of FimH to mannosylated proteins, such as A62, G66, and Y137, as well as those involved in the interdomain interactions [53,54,55,59]. Therefore, to assess whether prolonged d-mannose treatment might induce mutations on FimH critical residues altering its binding capacity to human mannosylated proteins *in vitro*, strain CFT073 was sub-cultured for 10 days in Luria Bertani (LB) supplemented with and without d-mannose at a final concentration of 1.5%. This exposure time was chosen because it is similar to that adopted by Domenici et al. for the treatment of acute UTIs [31]. After a further 48 h of incubation in static conditions [56,57], untreated, d-mannose-treated, and d-mannose-treated V-S bacteria were used to infect human bladder epithelial HTB-9 cells at a multiplicity of infection (MOI) of 10. Two hours post-infection, the number of cell-associated bacteria was calculated by CFU/mL counting (Figure 5). The d-mannose-treated V-S bacteria adhered as well as the untreated controls, while d-mannose treated bacteria showed a significantly lower extent of adhesion (Figure 5). Taken together, these results show that removal of d-mannose from FimH by applying mechanical forces left this adhesin fully proficient to bind to human urothelial mannosylated receptors. Overall, our results support the conclusion that the clinical regimen of d-mannose applied to treat acute UTIs (3 g/day for three days, then 1.5 g/day for 10 days, [31]) does not lead to FimH mutations that modify bacterial adhesiveness.

The effect of a high dosage of d-mannose on human epithelial cells was also evaluated. Semi-confluent HTB-9 monolayers were treated for 24 h with d-mannose at a final concentration of 1.5% (Figure S1). No macroscopic differences in the shape, integrity, adhesiveness, cytoplasmic vacuolization, proliferation, or cytotoxic effects were observed in HTB-9 cell monolayers incubated with d-mannose (Figure S1; data not shown). These results are in line with previous data showing no adverse side effects resulting from nonselective binding of some d-mannosides to human receptors [60,61].

## 3. Materials and Methods

### 3.1. Bacterial Strains, Yeast, and Cell Line

The well characterized uropathogenic *E. coli* strain CFT073 was kindly provided by Prof. U. Dobrindt and was used as uropathotype in this study. Strain CFT073 was grown at 37 °C in LB or seeded onto MacConkey agar plates. The presence of the *fimH* gene was confirmed by PCR using the primer pairs fimHF 5′-TGCAGAACGGATAAGCCGTGG-3′ and fimHR 5′-GCAGTCACCTGCCCTCCGGTA-3′ and using *E. coli fimH*-proficient and -deficient strains as positive and negative controls, strains E1P and I2P, respectively [62]. The yeast *S. cerevisiae* strain AH109 (Takara Bio, Ancona, Italy) was plated onto YPDA, as previously described [63]. The human bladder epithelial cell line 5637, HTB-9 (ATCC-LGC, Milan, Italy), was routinely cultured in T25 flasks at 37 °C in a humidified atmosphere with 5% CO_2_ using Roswell Park Memorial Institute (RPMI) 1640 medium (Gibco, Milan, Italy) supplemented with 10% FBS.

### 3.2. Culture Conditions, Reagents, and Bacterial Growth Measurements

Unless otherwise indicated, LB or MHA plates were supplemented with the following d-mannose concentrations 1.25, 2.5, 5, 10, 15, and 20% (Utismile^®^, S.I.I.T, Milan, Italy). LB and MHA plates were used as control. Swimming motility was assessed on LB soft agar plates. For some experiments, strain CFT073 was grown in M9 minimal medium (M9) (ThermoFisher, Milan, Italy), supplemented with d-mannose (Utismile^®^), d-glucose, d-fructose, and l-arabinose (Sigma Aldrich, Rome, Italy). From the overnight culture, bacteria were split to test different conditions and grown at 37 °C. Antibiotic disks were provided from Biomerieux (Florence, Italy). The growth kinetics of strain CFT073 growing in the different conditions were determined by optical density (OD_600_) and by colony-forming units (CFU) by spot-plating serial dilutions.

### 3.3. Measurements of Adenosine Triphosphate (ATP) Production

Equal amounts of bacteria normalized by OD_600_ were aliquoted into black 96 multiwell (Corning Costar^®^, ThermoFisher, Milan, Italy). The ATP measurements were performed using the BacTiter-Glo™ Microbial Cell Viability Assay Kit, following the manufacturer’s instruction (Promega, Milan, Italy). The luminescence produced by the Ultra-Glo™ Recombinant Luciferase was recorded on a GloMax^®^ 96 Microplate Luminometer (Promega, Milan, Italy).

### 3.4. Expression Levels of the ManX Permease

Total RNA was extracted from strain CFT073 grown in M9 minimal medium supplemented with either d-glucose, d-mannose, d-fructose, or l-arabinose grown to an OD_600_ value of 1.0 using the RiboPure™-Bacteria Kit (Ambion, ThermoFisher, Milan, Italy). The quantity and quality of the purified RNA were assessed by measuring the absorbance at OD_260_ and using formaldehyde agarose gel electrophoresis, respectively. cDNA was generated using PrimeScript™ RT Reagent Kit with a random primer hexamer mix and 1 µg of total RNA, following the manufacturer’s instructions (Takara Bio, Ancona, Italy). Semi-quantitative real-time PCR (RT-PCR) was performed in the Bio-Rad iQ5 real-time PCR detection system with iQ SYBR Green Supermix (Bio-Rad, Milan, Italy), using the following conditions: 5 min at 95 °C, 40 cycles at 95 °C for 15 s and 60 °C for 30 s. The sequences of the primer pairs used for *manX* were manXF 5′-GGGCCAAACGACTACATGGTTATT-3′ and manXR5′-ACCTGGGTGAGCAGTGTCTTACG-3′, and for the 16S rRNA gene (*rrsG*), used for data normalization, rrsGF 5′-GGTGTAGCGGTGAAATGCGTAG-3′ and rrsGR 5′-TCAAGGGCACAACCTCCAAGTC-3′. To determine the fold change expression of *manX* in different conditions the 2^−ΔΔCt^ method was used, as described previously [64], considering the bacteria grown in d-glucose as the reference condition.

Expression of ManX was assessed in whole cell extract (WCE) collected from bacteria grown under the same experimental conditions by Western blot. Equal amounts of WCE were loaded onto 12.5% Tris–glycine SDS-PAGE [65], transferred onto PVDF membranes (GE-Healthcare Bio-Sciences, Rome, Italy) and hybridized using polyclonal anti-ManX antibody (Abmart, Shanghai, China). The outer membrane protein A (OmpA) was used as a protein loading control, as reported elsewhere [64]. Blots were visualized by the enhanced chemiluminescence system (GE-Healthcare Bio-Sciences, Rome, Italy). Relative band intensities were quantified by densitometric analysis using the ImageJ software [66] and the ratio of ManX/OmpA was expressed as arbitrary units.

### 3.5. CFT073 Adhesion Assays

Confluent cell monolayers of human HTB-9 cells were infected with strain CFT073 grown in the absence and presence of 1.5% d-mannose at a multiplicity of infection (MOI) of 10, centrifuged and incubated at 37 °C with 5% CO_2_ for 2 h [56,57]. Cell monolayers were extensively washed to remove unbound bacteria, then lysed with Triton X-100 0.1% in PBS. Cell lysates were serially diluted and spot-plated onto LB agar plates for CFU/mL counting.

### 3.6. Agglutination of Yeast Cells

The binding activity of FimH was evaluated by yeast agglutination assay [56,57,67]. Strain CFT073 was grown in the absence and presence of 1.5% d-mannose for 48 h in static conditions. An aliquot of the bacterial culture grown in the presence of d-mannose was withdrawn, washed twice with PBS, vortexed at maximum speed for 20 min at room temperature and sonicated using an ultrasonic water bath, applying 35 kHz per 5 min in cold water (Fisherbrand™ S-Series, ThermoFisher, Milan, Italy). The vitality of vortexed and sonicated bacteria (referred to as V-S bacteria) was assessed by CFU/mL counting. Untreated, d-mannose-treated, and d-mannose-treated V-S bacteria were resuspended to an OD_600_ ≈ 1 and incubated individually with *S. cerevisiae* cells suspended in PBS to an OD_600_ ≈ 4 for 1 h at room temperature. Each suspension was spotted on a microscope slide and streaked for Gram-staining. Images were acquired both macroscopically and microscopically (10× and 40× magnifications).

### 3.7. Urine Glucose Measurements

Fifty-four urine samples from the clinical pathology unit, Umberto I University Hospital in Rome, previously tested as negative in routine microbiological tests were analyzed for glucose quantification using Glucose Colorimetric/Fluorometric Assay Kit MAK263, following the manufacturer’s instruction (Sigma Aldrich, Rome, Italy). Clinical samples used in this study were obtained during institutional diagnostic service; investigation described in this study could be carried out on residual specimens following diagnostic analysis provided that all data were kept anonymous.

### 3.8. Cell Viability and Proliferation (MTT test)

The viability and growth of HTB-9 cells was assessed by the MTT [3-(4,5-dimethyl-2-thiazolyl)-2,5-diphenyl-2H-tetrazolium bromide]-based assay, following manufacturer’s instructions (Abcam Ltd., Cambridge, UK). Images were acquired microscopically (40× magnification).

### 3.9. Statistical Analysis

Statistical differences were analyzed by one-way ANOVA test using the Bonferroni post-hoc test and unpaired t test. The values where *p* < 0.05 were taken as being statistically significant. Statistical data analysis was performed using GraphPad Prism software (version 5.01, La Jolla, CA, USA).

## 4. Conclusions

The first essential step for the establishment of the infection by UPEC is the steady adhesion to eukaryotic cells. UPEC adhesion relies mainly on the interaction between FimH and mannosylated uroplakin proteins on the luminal surfaces of urothelial cells as well as other cellular receptors such as integrins [1,5,8,13,14,15]. Therefore, FimH antagonists, such as d-mannose, have gained increasing consideration and proven to be effective for the treatment and/or prevention of acute and recurrent UPEC-mediated UTIs [21,22,24,25,26,27]. Furthermore, d-mannose represents a real alternative to antibiotic regimens, reducing the burden of antibiotic resistance and associated side effects [2,14,31,34,35,36]. Our results show that d-mannose affected neither bacterial viability, shape, or motility nor interfered with the activity of the tested antibiotics. Moreover, in the hierarchy of *E. coli* preferred sugars, d-mannose ranked as the least preferred in supporting the bacterial metabolism and growth and its usage was prevented even after 4 h of d-glucose consumption. Since our data showed that small amounts of glucose are normally present in urine from healthy subjects, we can conclude that the dosages of d-mannose used in clinical practice are irrelevant for *E. coli* metabolism and growth. Finally, d-mannose prolonged exposure did not select FimH variants that modify bacterial adhesiveness after d-mannose removal, further indicating that it does not exert “antibiotic-like” activity. Overall, the low metabolic/energetic advantages for bacterial growth, the lack of selection of altered FimH adhesins after long-term d-mannose exposure, and the bladder cell tolerance emphasize the safe use of d-mannose in the treatment and prevention of UTIs caused by UPEC.

## Supplementary Materials

The following are available online, Figure S1: Giemsa staining of HTB-9 cell monolayers incubated with 1.5% d-mannose for 24 h. Representative images of two independent experiments are shown. Scale bar, 10 μm. Images were recorded with the 40X objective using a Leica DM5000B microscope and processed using the Leica Application Suite 2.7.0.R1 software (Leica). Scale bar: 10 μm.
