# Supplementary material for: d-Mannose Treatment neither Affects Uropathogenic Escherichia coli Properties nor Induces Stable FimH Modifications
Source: Molecules. 2020 Jan 13;25(2):316. doi: 10.3390/molecules25020316 (PMC7024335; doi:10.3390/molecules25020316)
Supplement: Supplementary file 1 [file molecules-25-00316-s001.pdf]

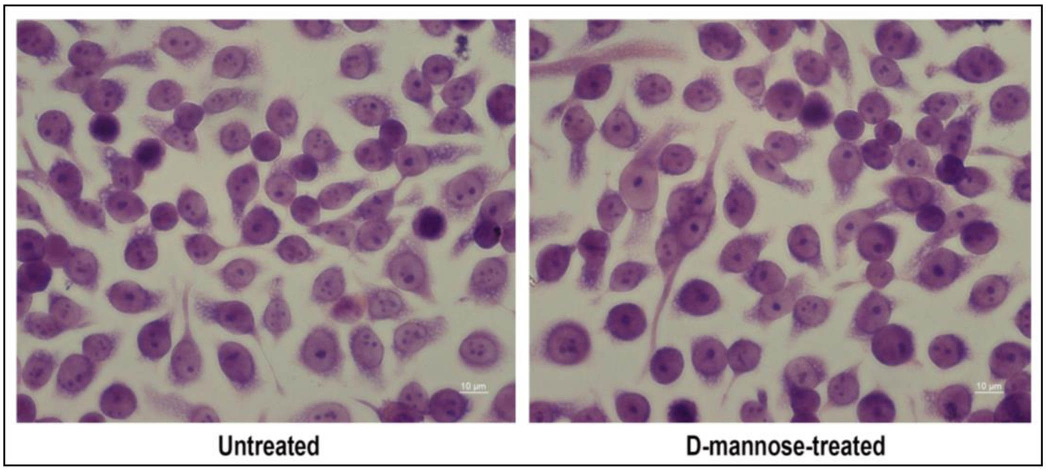

Figure S1. Giemsa staining of HTB-9 cell monolayers incubated with 1.5% D-mannose for 24 h. Representative images of two independent experiments are shown. Scale bar, 10 µm. Images were recorded with the 40X objective using a Leica DM5000B microscope and processed using the Leica Application Suite 2.7.0.R1 software (Leica). Scale bar: 10 µm.
